# COVID-19 Employment Crisis in Vietnam: Global Issue, National Solutions

**DOI:** 10.3389/fpubh.2020.590074

**Published:** 2020-12-04

**Authors:** Huong T. T. Nguyen, Tham T. Nguyen, Vu A. T. Dam, Long H. Nguyen, Giang T. Vu, Huong L. T. Nguyen, Hien T. Nguyen, Huong T. Le

**Affiliations:** ^1^School of Preventive Medicine and Public Health, Hanoi Medical University, Hanoi, Vietnam; ^2^Vietnam National University School of Medicine and Pharmacy, Vietnam National University, Hanoi, Vietnam; ^3^Center of Excellence in Evidence-Based Medicine, Nguyen Tat Thanh University, Ho Chi Minh City, Vietnam; ^4^Institute for Global Health Innovations, Duy Tan University, Da Nang, Vietnam; ^5^Faculty of Nursing, Duy Tan University, Da Nang, Vietnam

**Keywords:** SARS-CoV-2, economy, unemployment, solutions, Vietnam, COVID-19

Since the first case being reported in January 23, Vietnam has 911 confirmed cases (430 recovered and 460 actives) with 21 fatalities (1). A large number of people being infected and died from the disease, unprecedented measures taken by Vietnam government to curb the infection rate—from social distancing to locking down which involve business closing, have significantly affected economy, especially in terms of employment. Specifically, the groups of industries such as: Aviation services; Hotel/Food and Beverage Service and other services when revenue dramatically decreased about 50 and 23.6%, respectively, compared to the same period last year (2). According to the General Statistics Office of Vietnam, in the first 6 months of 2020, the number of enterprises suspending business for a definite time was 29.2 thousand, increasing by 38.2% over the same period last year, while 19.6 thousand enterprises stopped and focused mainly on the service sector. There were 897.5 thousand people losing jobs while the number of unemployed people was at 1.3 million, an increase of 123.9 thousand, making the unemployment rate reaching the highest point in 10 years (3). In which, the groups of non-working age groups, female workers, unskilled workers, migrant workers, and informal workers are the most vulnerable groups caused by pandemic (4, 5). In particularly, the female workers group, the unemployment rate was about 2.9%, higher than that of men and increased sharply over the same period last year (3) and for the informal workers—workers without labor contracts, unemployment insurance (4), there was 72% of those in the group affected by the COVID-19 pandemic when they mainly focused on Industry groups suffered the most damage: F&B, Hotel, logistic, etc. (6–8). In addition, domestic migrant workers, which account for 13.6% of the total population, often work in the informal economy without a work contract and without access to social protection regimes (8).

However, one thing is clear. With various forms of lockdowns and social distancing, Vietnam are facing a really difficult time. Out of 51.8 million employed workers in the second quarter of this year, 30.8 million people were affected by epidemics, of which 2.4 million workers lost their jobs, the national unemployment rate increase (7). In which, about 17.6 million people suffer income loss due to disease, accounting for 57.3% of the total affected people (9). COVID-19 pandemic directly impact employment. The number of employees in several occupations decreased a sharp fall compared to the last year as follows: unskilled group reduced by nearly 1.5 million workers, equivalent to nearly 8%; the group of craft and related trades workers decreased by 515 thousand persons, equivalent to a fall of 6.6%; the number of employees in the middle-level qualification group fell by 322 thousand persons, equivalent to a decrease of 16.5% (6). The General statistics office of Vietnam reported that the average monthly income of workers decreased for the first in 5 years. Especially, the deepest decrease in monthly average income in the 2nd quarter of 2020 compared to the same period last year as follows “Arts, entertainment and recreation,” “accommodation and catering service.” “transportation and storage” “wholesale and retail trade and repair of motor vehicles and motorcycles” down 19.2, 18.3, 12.8, and 9.1%, respectively (6). With unemployment rising and income per labor reduction has led to the economic growth rate only reached the level of 1.81% in the first 6 months of the year—the lowest figure recorded in the whole period 2011–2020 (10). The COVID-19 pandemic caused the income of many Vietnamese households to decrease by 70% (11). In addition, household income deepest decrease the most due to the Covid-19 epidemic, recorded in April 2020, when only 29.7% compared to December 2019. This figure to May 2020 is 51.1% (11). Children can also be affected by loss of a job or income loss from their parents (8). More than 21 million students in Viet Nam being affected by school closures (12). It is estimated that more than 1 million childrens aged 5–17 years are engaged in child labor, this children were face to work longer hours or in worse conditions (13). During the COVID-19 pandemic, dropouts, malnutrition, labor exploitation, and child labor can increase dramatically. This lead to serious consequences, especially for the development of the workforce.

With the negative impact of COVID-19 pandemic, Vietnam's economic growth in 2020 also was decreased by about 4% compared to last year (14, 15). However, the Vietnam government had several solutions manage the fiscal deficit for solving immediate problems such as focus on effectively implementing domestic stimulus, use the savings from falling international oil prices to curb the crisis, earn funding from the World Bank WB and the International Monetary Fund, etc. (16, 17). Besides, economic recovery solutions such as promoting production, business, socio-economic development are also offered in three main contents: General solutions; urgent solutions; and Long-term solutions (Figure 1). Firstly, according to The Politburo on the policy of overcoming the impact of the COVID-19 pandemic to improve and develop the economy, the Government would provide support in the form of (1) to make the most of the domestic market, at the same time to prevent and respond to instabilities from outside; (2) to develop a favorable and attractive business environment, suitable to new trends, and have regional and international competitiveness; (3) to identify opportunities and challenges to take advantage of solutions, transform opportunities, and challenges into motivation on economic growth (18). Secondly, for urgent solutions for solving immediate problems consist of (1) extension for payment of tax and other duties (social insurance, trade union fees, etc.); (2) financial assistance through policies that require lending institutions to reduce lending rate, facilitate debt rescheduling, provide liquidity for businesses affected by COVID-19; (3) monetary allowance and 0% interest loan for those having their employment affected by the pandemic; and (4) promoting of the domestic market and stimulating domestic consumption (19–21). In addition, some solutions such as Stop paying social insurance, stop paying premiums, reduce union fee by 50% and reduce 15% land rent reduction policy should be applied to all businesses because most of the businesses are affected, possibly operating but facing many difficulties (according to Resolution 84). Stabilize electricity and water prices of enterprises and eliminate monopoly prices in this field. A number of policies on banking and finance such as solutions to support liquidity, debt rescheduling, and debt group retention; Reduce lending interest rates; Extending credit guarantee measures so that small, medium and micro-enterprises, business households can borrow capital (22). Specifically, the Vietnamese government has introduced a US $ 2.7 billion relief fund to support all workers affected by the COVID-19 pandemic in Vietnam. In particular, this bailout package focus on support for businesses in difficulty, workers deeply affected by income, and vulnerable people who are not supported to access many existing social security networks within 3 months (April, May, and June) with different levels of support for each group of workers (23). Finally, for long-term solution, Vietnam's government have strategies include (1) developing policies and legislation to facilitate new business models; (2) economic restructuring and support potential and advantageous industries; (3) improving competitiveness and support businesses/enterprise; (4) innovating, creating, applying science and technology; (5) meeting a need of social security, employment, and human resources training; and (6) developing and strengthening key economic regions. Vietnam has drastically rectified the job crisis related to COVID-19 and most importantly, minimizes the damage that affects businesses and employees. Therefore, it is important to ensured that the socio-economic policy is built based on three aspects: the Government, employers, and workers. Vietnam has dealt with the job crisis related to COVID-19 drastically. It is important to minimize damage affecting businesses and workers. This is an important time to ensure that the socio-economic policy is built in an inclusive manner based on government, employers, and workers. These difficult times provide an opportunity for Vietnam to establish a more inclusive growth platform.

**Figure 1:**
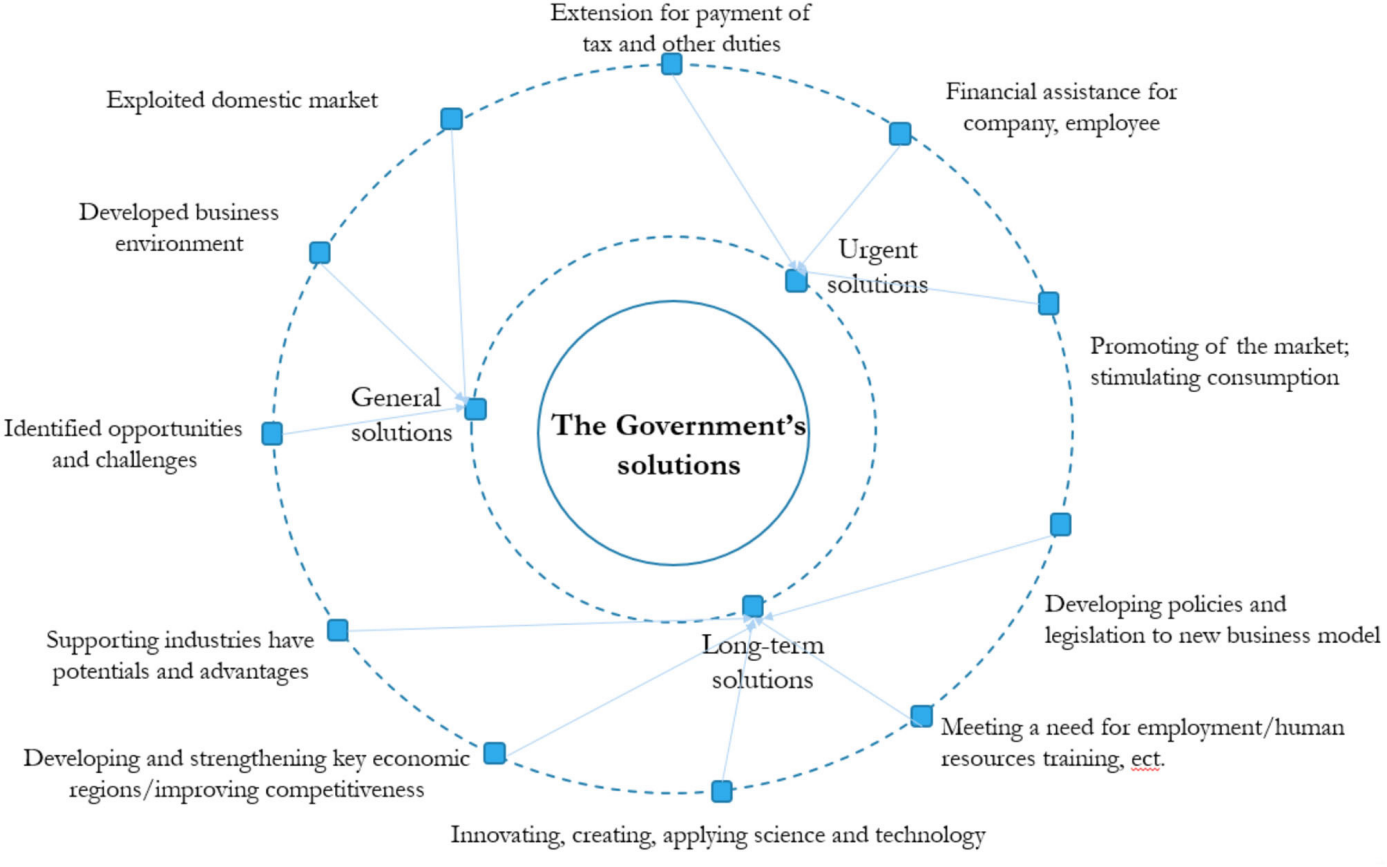
The diagram of the Government's solutions to support the unemployed.

In COVID-19 pandemic, Vietnam faces many opportunities and challenges for the businesses as well as workers in the process of adapting to the new economic model. Generally, the business market suffered heavy losses due to the crisis, which led to an inordinate increase in the unemployment rate (2.73%) (7). In terms of urgent period, there are several challenges for the economy when market demand decreased and becomes more competitive due to some industries are strongly affected such as the GDP of export industry and services decreased about 8.45–9.67% (7). This also leads to the highest unemployment rate in the 10 years and a concentration of a large number of vulnerable labor (6). Therefore, business owners need to grasp the demand for their products/services as well as the availability of labor force during and after the pandemic, to identify new opportunities and implement timely business model transformation. Additionally, it requires the Government and business to offer several measures to support workers especially the vulnerable group: financial support and job hunting (23, 24). Besides the challenges, some policies have been introduced to support businesses to maintain business, retain employees, and minimize employee layoffs (25). Specifically, businesses are entitled to tax extension, tax reduction, and financial support (22). Therefore, the risk of unemployment and income reductions can be reduced, especially in sectors such as services and import-export which are most affected (26). In the long term, businesses can be affected for a long time and the business market in Vietnam needs time to recover. Key sectors such as service and import-export still suffer due to the disease's complicated evolution of the epidemic and the mandatory quarantine policy upon entry in Vietnam (According to Official dispatch 1440 of the National steering committee for Disease Control and Prevention COVID-19). Vietnam implemented a social division from April 1 to April 23 (According to Directive 15 of the National steering committee for Disease Control and Prevention COVID-19). This lead to several opportunities and challenges for companies and employees. Specifically, businesses need to develop existing markets and exploit new markets such as developing the domestic market when import-export is limited, and prioritize the development of the online transaction market. From there, it is possible to form the habit of prioritizing local consumption for Vietnamese customers. Development of the digital era and technological revolution 4.0 is the big chance for all enterprise/business. It contributes to improving production capacity, promoting products, creating potential opportunities in implementing online sales services which are a strong development model during the epidemic. Finally, it is important that businesses proactively come up with the right solutions for themselves to adapt to new markets and employees also actively seek opportunities, hone their skills to adapt to new business model of the market.

## Author Contributions

HTTN, TTN, VATD, LHN, GTV, and HTL: conceptualization. HTTN, TTN, and VATD: writing original draft. HTTN, TTN, VATD, LHN, GTV, HTL, HTN, and HLTN: writing, review and editing. LHN and GTV: project administration. All authors contributed to the article and approved the submitted version.

## Conflict of Interest

The authors declare that the research was conducted in the absence of any commercial or financial relationships that could be construed as a potential conflict of interest.
